# Supplementary material for: Rapid single-tier serodiagnosis of Lyme disease
Source: Nat Commun. 2024 Aug 20;15:7124. doi: 10.1038/s41467-024-51067-5 (PMC11336255; doi:10.1038/s41467-024-51067-5)
Supplement: Supplementary file 1 — Supplementary Information [file 41467_2024_51067_MOESM1_ESM.pdf]

Supplementary Information: Rapid single-tier serodiagnosis of Lyme disease

Rajesh Ghosh, Hyou-Arm Joung, Artem Goncharov, Barath Palanisamy, Kevin Ngo, Katarina Pejcinovic, Nicole Krockenberger, Elizabeth J. Horn, Omai B. Garner, Ezdehar Ghazal, Andrew O'Kula8, Paul M. Arnaboldi, Raymond J. Dattwyler, Aydogan Ozcan* and Dino Di Carlo*

*Corresponding Authors: ozcan@ucla.edu ; dicarlo@ucla.edu

Contents

- Supplementary Table 1: Anti-human IgM and IgG antibodies screened.
- Supplementary Table 2: Synthetic peptide antigens screened.
- Supplementary Table 3: Bay Area Lyme Foundation's Lyme disease biobank samples used in training of xVFA.
- Supplementary Table 4: CDC's Lyme serum repository samples used for blinded validation of xVFA.
- Supplementary Table 5: Comparison of cost for xVFA test using recombinant protein antigens and synthetic peptides.
- Supplementary Figure S1: Stability of the combined peptide synthetic panel and machine learning diagnostic algorithm.
- Supplementary Figure S2: Optimization of secondary antibodies.
- Supplementary Figure S3: Screening of BBA64-7, BBA65-94, BBA73 196-199 peptides using ELISA.
- Supplementary Figure S4: Sensitivity and specificity of individual peptides selected in the multiplexed panel.
- Supplementary Figure S5: Optimization of the xVFA to improve signal to noise ratio by tuning the buffer composition, IgM and IgG antibodies, sensing membrane porosity, AuNP size and sample volume.

Supplementary Table 1. Anti-human secondary IgM and IgG antibodies screened for binding against anti-*Borrelia* antibodies for the detection of LD infection.

Code	Vendor	Host	Type	CAT#	Antibody
G1	abcam	Mouse	Monoclonal	ab99770	Mouse monoclonal [H2] Anti-Human IgG Fc
G2	SouthernBiotech	Mouse	Monoclonal	9040-01	Mouse Anti-Human IgG Fc-UNLB (JDC-10)
G3		Mouse	Monoclonal	9042-01	Mouse Anti-Human IgG Fc-UNLB (H2)
G4		Goat	Polyclonal	2014-01	Goat Anti-Human IgG Fc, Multi-Species SP ads-UNLB

M1	abcam	Mouse	Monoclonal	ab99741	Mouse monoclonal UHB Anti-Human IgM mu chain
M2	SouthernBiotech	Mouse	Monoclonal	9020-01	Mouse Anti-Human IgM-UNLB (SA-DA4)
M3		Mouse	Monoclonal	9022-01	Mouse Anti-Human IgM-UNLB (UHB)
M4		Goat	Polyclonal	2023-01	Goat Anti-Human IgM, Mouse/Bovine/Horse SP ads-UNLB

Supplementary Table 2. List of all synthetic peptide antigens and their amino acid sequences used in this study.

Synthetic peptide name	Amino acid sequence	Strain	Molecular weight
OspC	MTLFLFISCNNSGKDGNTSAC	B31	2223.53
OppA	CYGQNWTSPENMVTSGPFKLKERIPNEKYVFEKNNK	B31	4277.88
p35	CDTGSERSIRYRRRVY	B31	2017.17
ErpP	CKIEFSKFTVKIKNKD	B31	1928.33
DbpA	CTILVNLLISCGLTGA	B31	1590.96
DbpB	CKDLKNKILKIKKEATGKGVLFEAFTGLKTG	B31	3380.12
BBK32	CKKPMNKKGKGKIARKKGKSKVSRKEPYIHS	B31	3541.36
BBA64	CNESKKHKKEKRKGKV	B31	1927.31
BBA65-94	CTIKKISGGIRIQQGFEVA	B31	2048.45
BBA65-101-02	CKNTLEEIYNLIVDLTLIKKEW	B31	2679.19
RecA164	CIMFINQIRMRIGVMFGNPETTT	B31	2673.25
RecA168-69	CGGNALKFYSSLRLEVRKIEQVTR	B31	2768.25
LA-7	CIPSKENAKLIVYFYDNVYAG	B31	2407.78
FlilB4	CVSRKGGLLPDIIIKI	B31	1725.18
modVlsE-FlaB	CVQEGVQQ EGAQQPGGGMKKNDQ IVAAIALRGVA	B31	3451.95
Var2FlaB	CMKKTDNIAAAIVIRGVAKDGQFALKGGGVQEGVQQEGAQQP	B31	4312.97
DbpA4-B6	TILVNLLISCGLTGAGGGGGKDLKNKILKIKKEATGKGVLFEAFTGLKTGC	B31	5135.19
BBA73-196-99	MKRNKIWKTLKLFQITLLFSCSFYSKSNNTC	B31	3743.75

Supplementary Table 3. Clinical samples and corresponding ground truth data from Cohort 3 of the Lyme Disease Biobank used to train the single-tier xVFA.

	Clinical presentations		*Reference tests*				*First-tier tests*			*Second-tier tests*			
**Sample #**	***EM/ Annular Rash***	***EM > 5 cm at Enrollment***	***B. burgdorferi PCR***	***B. miyamotoi PCR***	***Anaplasma PCR***	***B. Microti PCR***	***Whole Cell Lysate ELISA***	***C-6 Peptide ELISA***	***VlsE/PepC 10 ELISA***	***Western Blot IgM****	***Western Blot IgG#***	***Two-tier Positive***	***xVFA prediction (triplicate)***
LDB01	YES	NO	NEG	NEG	NEG	NEG	POS	POS	NA	POS	EQV	YES	
LDB04	YES	NO	NEG	NEG	NEG	NEG	NEG	POS	NA	POS	EQV	YES	
LDB05	NO	NA	NEG	NEG	NEG	NEG	POS	POS	NA	POS	EQV	YES	
LDB07	YES	YES	NEG	NEG	NEG	NEG	NEG	POS	NA	POS	POS	YES	
LDB08	NO	NA	NEG	NEG	NEG	NEG	NEG	POS	NA	POS	EQV	YES	
LDB10	YES	NO	NEG	NEG	NEG	NEG	EQV	POS	NA	POS	EQV	YES	
LDB11	YES	YES	NEG	NEG	NEG	NEG	NA	POS	POS	POS	NEG	YES	
LDB12	NO	NA	NEG	NEG	NEG	NEG	NA	EQV	POS	POS	NEG	YES	
LDB13	NO	NA	NEG	NEG	NEG	NEG	NA	NEG	EQV	POS	NEG	YES	
LDB14	YES	YES	NEG	NEG	NEG	NEG	NA	POS	POS	POS	NEG	YES	
LDB15	NO	NA	NEG	NEG	NEG	NEG	NA	POS	POS	POS	NEG	YES	
LDB16	YES	YES	NEG	NEG	NEG	NEG	NA	POS	NA	NEG	POS	YES	
LDB17	YES	NO	NEG	NEG	NEG	NEG	NA	NEG	POS	POS	NEG	YES	
LDB19	NO	NA	NEG	NEG	NEG	NEG	NA	NEG	POS	POS	NEG	YES	
LDB20	NO	NA	NEG	NEG	NEG	NEG	NA	POS	POS	POS	POS	YES	
LDB21	YES	YES	NEG	NEG	NEG	NEG	NA	POS	POS	POS	NEG	YES	
LDB22	YES	YES	NEG	NEG	NEG	NEG	NA	POS	POS	POS	POS	YES	
LDB23	NO	NA	NEG	NEG	NEG	NEG	NA	POS	POS	POS	POS	YES	
LDB24	YES	YES	NEG	NEG	NEG	NEG	NA	POS	POS	POS	NEG	YES	
LDB25	YES	YES	NEG	NEG	NEG	NEG	NA	POS	NA	POS	NEG	YES	
LDB36	NO	NA	NEG	NEG	NEG	NEG	EQV	NEG	NA	EQV	EQV	NO	
LDB37	NO	NA	NEG	NEG	NEG	NEG	NEG	NEG	NA	NEG	EQV	NO	
LDB38	NO	NA	NEG	NEG	NEG	NEG	NEG	NEG	NA	NEG	NEG	NO	
LDB40	NO	NA	NEG	NEG	NEG	NEG	NEG	NEG	NA	EQV	EQV	NO	
LDB41	NO	NA	NEG	NEG	NEG	NEG	NEG	NEG	NA	EQV	EQV	NO	
LDB42	NO	NA	NEG	NEG	NEG	NEG	NEG	NEG	NA	EQV	EQV	NO	
LDB43	NO	NA	NEG	NEG	NEG	NEG	NEG	NEG	NA	NEG	EQV	NO	
LDB44	NO	NA	NEG	NEG	NEG	NEG	NEG	NEG	NA	EQV	EQV	NO	
LDB46	NO	NA	NEG	NEG	NEG	NEG	NA	NEG	NEG	NEG	NEG	NO	
LDB47	NO	NA	NEG	NEG	NEG	NEG	NA	NEG	EQV	NEG	NEG	NO	
LDB48	NO	NA	NEG	NEG	NEG	NEG	NA	NEG	NEG	NEG	NEG	NO	
LDB49	NO	NA	NEG	NEG	NEG	NEG	NA	NEG	NEG	NEG	NEG	NO	
LDB50	NO	NA	NEG	NEG	NEG	NEG	NA	NEG	NEG	NEG	NEG	NO	
LDB51	NO	NA	NEG	NEG	NEG	NEG	NA	NEG	NA	NEG	NEG	NO	
LDB52	NO	NA	NEG	NEG	NEG	NEG	NA	NEG	NA	NEG	NEG	NO	
LDB54	NO	NA	NEG	NEG	NEG	NEG	NA	NEG	NEG	NEG	NEG	NO	
LDB55	NO	NA	NEG	NEG	NEG	NEG	NA	NEG	NEG	NEG	NEG	NO	
LDB56	NO	NA	NEG	NEG	NEG	NEG	NA	NEG	NEG	NEG	NEG	NO	
LDB58	NO	NA	NEG	NEG	NEG	NEG	NA	NEG	NEG	NEG	NEG	NO	
LDB59	NO	NA	NEG	NEG	NEG	NEG	NA	NEG	NEG	NEG	NEG	NO	

*Minimum of 2 of the 3 CDC specific bands (23,39,41 Kda) must be present.

#Minimum of 5 of the 10 CDC specific bands (18,23,28,30,39,41,45,58,66,93 Kda) must be present.

¶Blue indicates positive and grey indicates negative outcomes. Each sample was tested in triplicate, and all replicates are shown.

Supplementary Table 4. Clinical samples and corresponding ground truth data from Cohort 3 of the Lyme Disease Biobank used to validate the single-tier xVFA.

	Clinical presentations		*Reference tests*				*First-tier tests*			*Second-tier tests*			
Sample #	*EM/ Annular Rash*	*EM > 5 cm at Enrollment*	*B. burgdorferi PCR*	*B. miyamotoi PCR*	*Anaplasma PCR*	*B. Microti PCR*	*Whole Cell Lysate ELISA*	*C-6 Peptide ELISA*	*VlsE/PepC 10 ELISA*	*Western Blot IgM**	*Western Blot IgG#*	*Two-tier Positive*	*xVFA prediction (triplicate testing)¶*
LDB02	YES	YES	NEG	NEG	NEG	NEG	NEG	POS	NA	POS	EQV	YES	
LDB03	YES	YES	NEG	NEG	NEG	NEG	NEG	POS	NA	POS	EQV	YES	
LDB06	YES	YES	NEG	NEG	NEG	NEG	RE	POS	NA	POS	EQV	YES	
LDB09	YES	YES	NEG	NEG	NEG	NEG	EQV	POS	NA	POS	EQV	YES	
LDB18	YES	YES	NEG	NEG	NEG	NEG	NA	POS	POS	POS	POS	YES	
LDB26	NO	NA	NEG	NEG	NEG	NEG	NA	POS	POS	POS	NEG	YES	
LDB27	YES	YES	NEG	NEG	NEG	NEG	NA	POS	POS	POS	NEG	YES	
LDB28	YES	YES	NEG	NEG	NEG	NEG	NA	POS	POS	POS	POS	YES	
LDB29	YES	YES	NEG	NEG	NEG	NEG	NA	POS	POS	POS	NEG	YES	
LDB30	YES	YES	NEG	NEG	NEG	NEG	NA	POS	POS	POS	NEG	YES	
LDB31	NO	NA	NEG	NEG	NEG	NEG	NA	POS	POS	POS	NEG	YES	
LDB32	YES	YES	NEG	NEG	NEG	NEG	NA	POS	POS	POS	POS	YES	
LDB33	YES	YES	NEG	NEG	NEG	NEG	NA	POS	POS	POS	NEG	YES	
LDB34	YES	YES	NEG	NEG	NEG	NEG	NA	POS	POS	POS	NEG	YES	
LDB35	YES	YES	NEG	NEG	NEG	NEG	NEG	POS	POS	POS	NEG	YES	
LDB39	NO	NA	NEG	NEG	NEG	NEG	NEG	NEG	NA	NEG	NEG	NO	
LDB45	NO	NA	NEG	NEG	NEG	NEG	NEG	NEG	NA	EQV	EQV	NO	
LDB53	NO	NA	NEG	NEG	NEG	NEG	NA	NEG	NEG	NEG	NEG	NO	
LDB57	NO	NA	NEG	NEG	NEG	NEG	NA	NEG	NEG	NEG	NEG	NO	
LDB60	NO	NA	NEG	NEG	NEG	NEG	NA	NEG	NEG	NEG	NEG	NO	
LDB61	NO	NA	NEG	NEG	NEG	NEG	NEG	NEG	NA	EQV	EQV	NO	
LDB62	NO	NA	NEG	NEG	NEG	NEG	NEG	NEG	NA	EQV	NEG	NO	
LDB63	NO	NA	NEG	NEG	NEG	NEG	NA	NEG	NA	NEG	NEG	NO	
LDB64	NO	NA	NEG	NEG	NEG	NEG	NA	NEG	NEG	NEG	NEG	NO	
LDB65	NO	NA	NEG	NEG	NEG	NEG	NA	NEG	NEG	NEG	NEG	NO	
LDB66	NO	NA	NEG	NEG	NEG	NEG	NA	NEG	NEG	NEG	NEG	NO	
LDB67	NO	NA	NEG	NEG	NEG	NEG	NA	NEG	NEG	NEG	NEG	NO	
LDB68	NO	NA	NEG	NEG	NEG	NEG	NA	NEG	NEG	NEG	NEG	NO	
LDB69	NO	NA	NEG	NEG	NEG	NEG	NA	NEG	NEG	NEG	NEG	NO	
LDB70	NO	NA	NEG	NEG	NEG	NEG	NA	NEG	NEG	NEG	NEG	NO	

*Minimum of 2 of the 3 CDC specific bands (23,39,41 Kda) must be present.

#Minimum of 5 of the 10 CDC specific bands (18,23,28,30,39,41,45,58,66,93 Kda) must be present.

¶Blue indicates positive and grey indicates negative outcomes. Each sample was tested in triplicate, and all replicates are shown.

Supplementary Table 5. Cost breakdown of xVFA test using recombinant proteins and peptide-based antigen panel.

<table>
<tr><th rowspan="2">Types</th><th rowspan="2">Name</th><th colspan="2">Cost/test ($)</th></tr>
<tr><th>Antigen assay</th><th>Peptide assay</th></tr>
<tr><td rowspan="3">Paper materials</td><td>Asymmetric membrane</td><td colspan="2">0.38</td></tr>
<tr><td>Interpad</td><td colspan="2">0.07</td></tr>
<tr><td>NC membrane</td><td colspan="2">0.17</td></tr>
<tr><td colspan="2">Catridge (3D printed)</td><td colspan="2">1.00</td></tr>
<tr><td rowspan="8">Bio/chemical resources</td><td>Gold nanoparticles</td><td colspan="2">0.20</td></tr>
<tr><td>Anti-Human IgG</td><td colspan="2">0.17</td></tr>
<tr><td>Anti-Human IgM</td><td colspan="2">0.17</td></tr>
<tr><td>Anti-Mouse IgG</td><td colspan="2">0.002</td></tr>
<tr><td>BSA</td><td colspan="2">0.03</td></tr>
<tr><td>Others</td><td colspan="2">0.40</td></tr>
<tr><td>Antigens</td><td>10.4</td><td></td></tr>
<tr><td>Peptides</td><td></td><td>0.32</td></tr>
<tr><td></td><td>Total cost per test</td><td>12.99</td><td>2.59</td></tr>
</table>

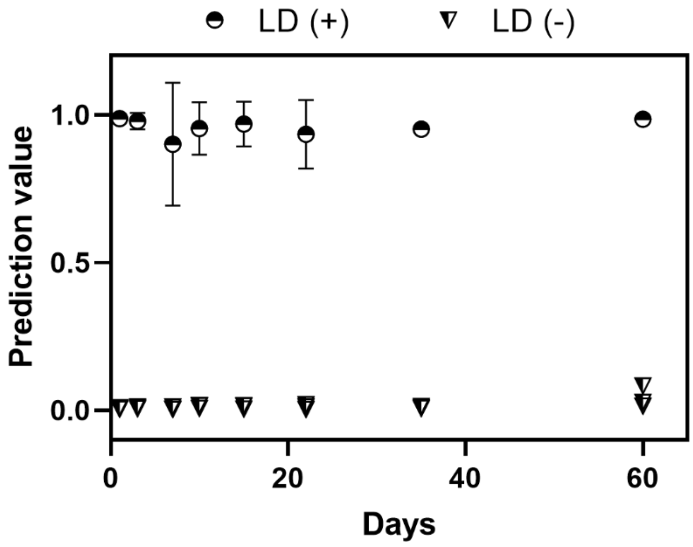

Supplementary Figure 1. The stability of the combined synthetic peptide panel was assessed over a period of 60 days, using both LD-positive and healthy control samples as benchmarks. The resulting plot illustrates the mean predictive values generated by the trained machine-learning diagnostic model for each of the xVFA assays throughout the testing duration (N=3). Data are presented as mean values ± SD.

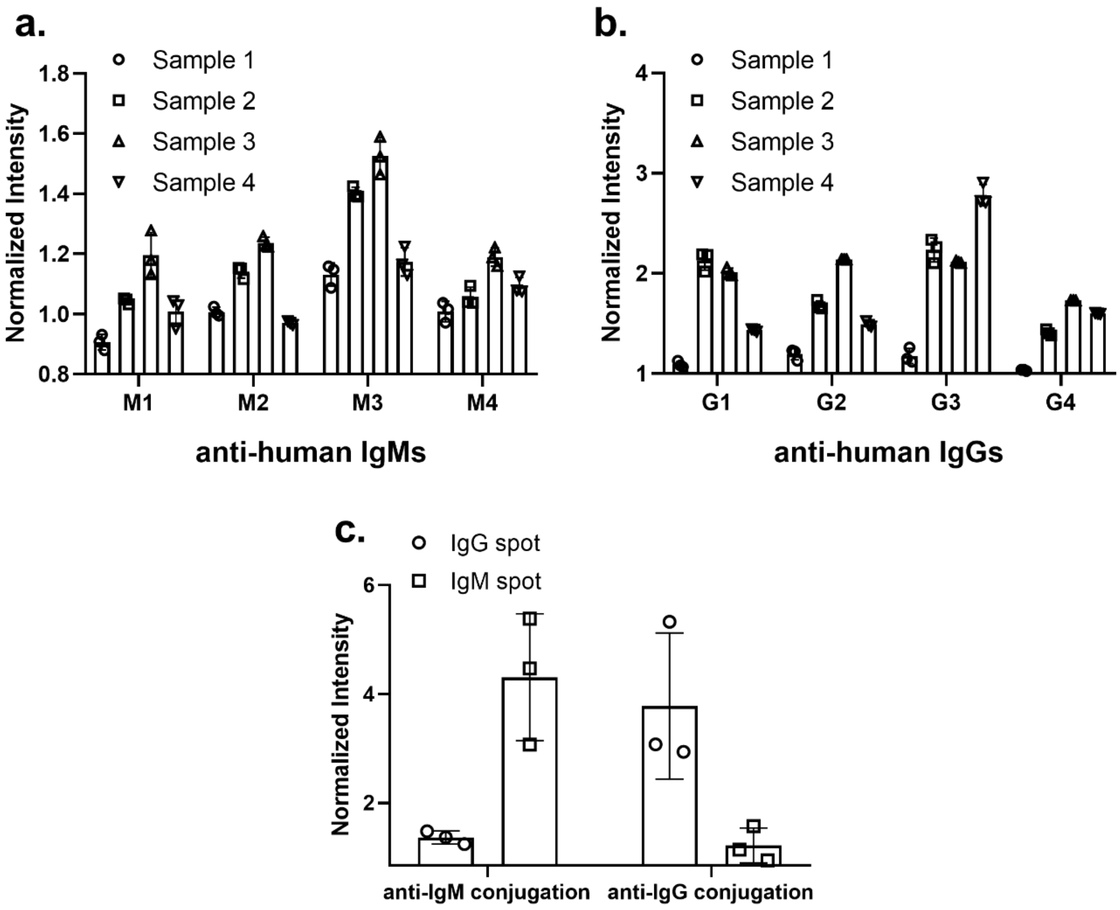

Supplementary Figure 2. Screening of anti-human antibodies for binding to Lyme disease **a** IgM and **b** IgG antibodies. **c** Cross-reactivity evaluation of the IgM and IgG antibodies selected in the detection of a control LD patient sample containing anti-*Borrelia* IgM and IgG antibodies. Data are presented as mean values ± SD, with three replicates for each (N=3).

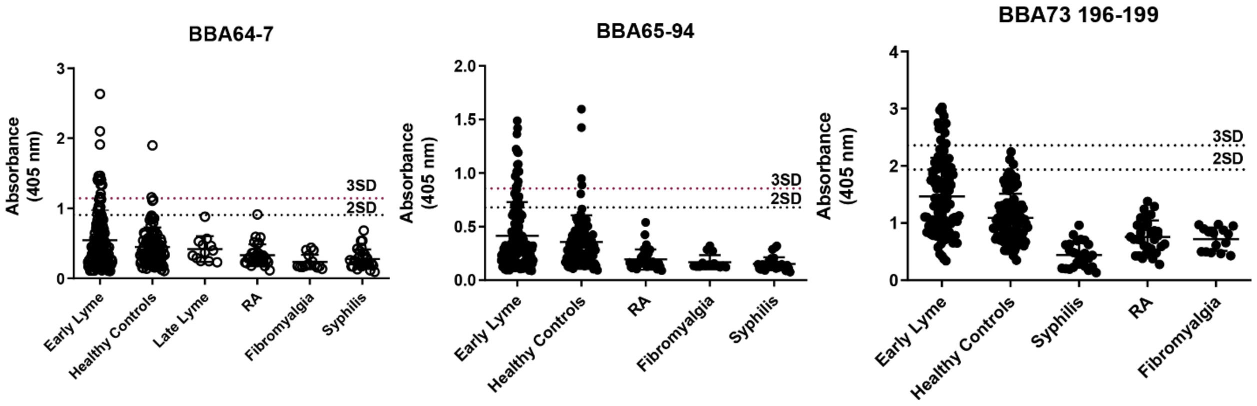

Supplementary Figure 3. Screening of BBA64-7, BBA65-94, BBA73 196-199 peptides using ELISA to measure interaction of synthesized peptides with anti-*Borrelia* IgM antibodies tested against sample groups containing early Lyme disease patient samples, late Lyme disease, healthy control groups and cross-reactive diseases such as rheumatoid arthritis, fibromyalgia and syphilis. The cut-off for positivity was defined as three times the standard deviation of the healthy controls and an equivocal result was defined as a signal above two times the standard deviation. If the absorbance recorded for a healthy control sample was above three times the standard deviation, it was not considered for the cut-off determination.

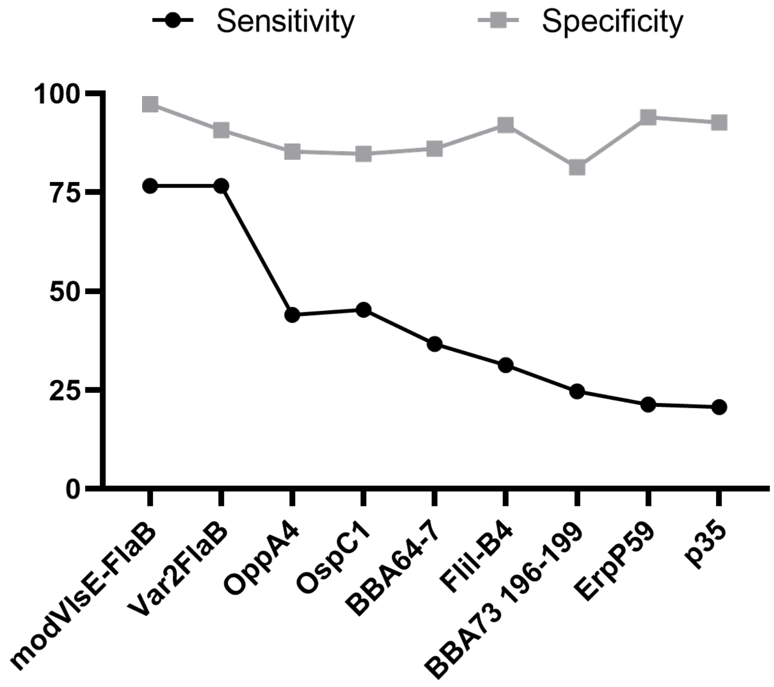

Supplementary Figure 4. Sensitivity and specificity of individual synthetic peptides selected for diagnosing LD using xVFA without combining the signal intensities from reaction spots using a neural network model.

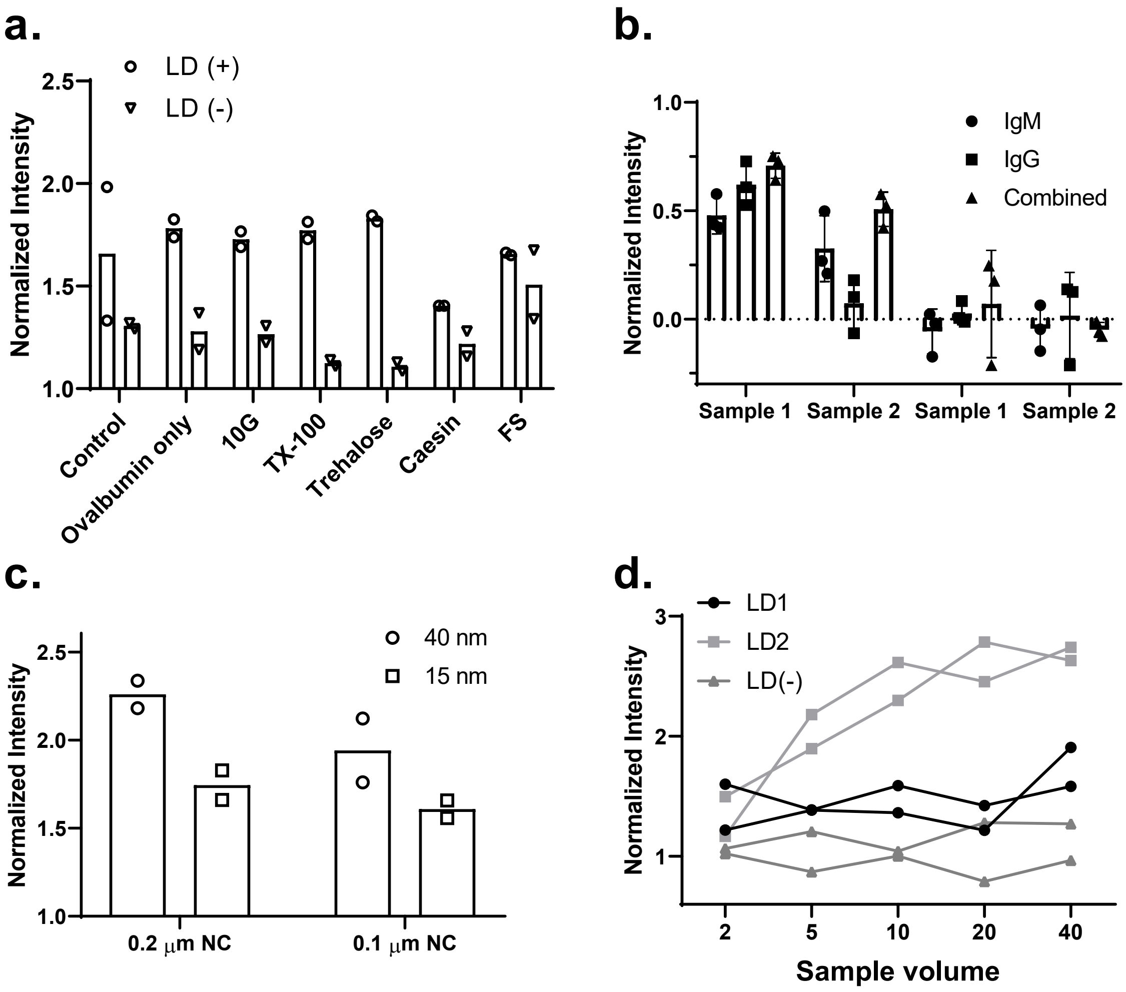

Supplementary Figure 5. Optimization of the xVFA operation and assay reagents. **a** Evaluation of different running buffer condition where control represents a buffer containing 2% (w/v) ovalbumin, and 3% (w/v) Tween-20. Other compositions tested along with the control buffer were ovalbumin without the Tween-20 surfactant, 3% (w/v) 10G surfactant, 1% (w/v) Triton X-100, 2% (w/v) trehalose, 2% (w/v) casein and 10x dilution of Thermofisher Scientific superblock® buffer. Buffer containing 2% (w/v) ovalbumin, 3% (w/v) Tween-20 and 2% (w/v) trehalose was found optimal. **b** Testing of control Lyme disease patient samples with gold nanoparticles labelled with IgM only, IgG only and combined IgM and IgG secondary antibodies. Data are presented as mean values ± SD (N=3). **c** Screening of deposition of 15 nm and 40 nm gold nanoparticles on 0.2 μm and 0.1 μm nitrocellulose membrane. 40 nm gold nanoparticles deposited on 0.2 μm nitrocellulose membrane was found optimal. **d** Optimization of the sample volume added during the xVFA operation using two control Lyme disease patient samples and one control healthy sample. The signal was almost saturated at 10 μL sample volume and 20 μL was chosen as the volume of sample added for all clinical tests carried out.
