# Supplementary material for: Rapid single-tier serodiagnosis of Lyme disease
Source: Nat Commun. 2024 Aug 20;15:7124. doi: 10.1038/s41467-024-51067-5 (PMC11336255; doi:10.1038/s41467-024-51067-5)
Supplement: Supplementary file 3 — Reporting Summary [file 41467_2024_51067_MOESM3_ESM.pdf]

nature portfolio

Corresponding author(s): Dino Di Carlo, Aydogan Ozcan

Last updated by author(s): Jul 27, 2024

Reporting Summary

Nature Portfolio wishes to improve the reproducibility of the work that we publish. This form provides structure for consistency and transparency in reporting. For further information on Nature Portfolio policies, see our Editorial Policies and the Editorial Policy Checklist.

Statistics

For all statistical analyses, confirm that the following items are present in the figure legend, table legend, main text, or Methods section.

n/a	Confirmed	
☐	☒	The exact sample size (*n*) for each experimental group/condition, given as a discrete number and unit of measurement
☐	☒	A statement on whether measurements were taken from distinct samples or whether the same sample was measured repeatedly
☐	☒	The statistical test(s) used AND whether they are one- or two-sided<br>*Only common tests should be described solely by name; describe more complex techniques in the Methods section.*
☒	☐	A description of all covariates tested
☒	☐	A description of any assumptions or corrections, such as tests of normality and adjustment for multiple comparisons
☐	☒	A full description of the statistical parameters including central tendency (e.g. means) or other basic estimates (e.g. regression coefficient) AND variation (e.g. standard deviation) or associated estimates of uncertainty (e.g. confidence intervals)
☐	☒	For null hypothesis testing, the test statistic (e.g. *F*, *t*, *r*) with confidence intervals, effect sizes, degrees of freedom and *P* value noted<br>*Give P values as exact values whenever suitable.*
☒	☐	For Bayesian analysis, information on the choice of priors and Markov chain Monte Carlo settings
☒	☐	For hierarchical and complex designs, identification of the appropriate level for tests and full reporting of outcomes
☒	☐	Estimates of effect sizes (e.g. Cohen's *d*, Pearson's *r*), indicating how they were calculated

Our web collection on statistics for biologists contains articles on many of the points above.

Software and code

Policy information about availability of computer code

Data collection	Images of xVFA were captured using the Smartphone based reader in DWG format.
Data analysis	Images were processed using Python 3.11.4. Data were analyzed with Microsoft Excel for Microsoft 365 MSO (Version 14.0.16731.20182), MATLAB 2022b and GraphPad Prism 8.0.1

For manuscripts utilizing custom algorithms or software that are central to the research but not yet described in published literature, software must be made available to editors and reviewers. We strongly encourage code deposition in a community repository (e.g. GitHub). See the Nature Portfolio guidelines for submitting code & software for further information.

Data

Policy information about availability of data

All manuscripts must include a data availability statement. This statement should provide the following information, where applicable:

- Accession codes, unique identifiers, or web links for publicly available datasets
- A description of any restrictions on data availability
- For clinical datasets or third party data, please ensure that the statement adheres to our policy

All experiment data and supporting findings are presented in the paper and Supplementary Information in graphic and table form. Source data are provided with this paper.

Research involving human participants, their data, or biological material

Policy information about studies with human participants or human data. See also policy information about sex, gender (identity/presentation), and sexual orientation and race, ethnicity and racism.

Reporting on sex and gender	De-identified human samples acquired from the Lyme Disease Biobank used for training and testing of the assay had a nearly even gender distribution, with 48.3% females and 51.3% males.
Reporting on race, ethnicity, or other socially relevant groupings	The study categorized participants based on self-reported race and ethnicity data. Categories included White, African American, Hispanic, Asian, and other categories such as American Indian or Alaska Native. Definitions were provided by the participants through self-report during initial data collection. Confounding variables were controlled by stratifying the analysis by race and ethnicity to ensure comprehensive evaluation.
Population characteristics	De-identified human samples acquired from the Lyme Disease Biobank included a diverse cohort with various stages of Lyme disease, including early, disseminated, and late-stage. Samples were from endemic regions and non-endemic areas. Participants ranged in age from 18 to over 89 years, with 48.3% females and 51.3% males. Ethnic backgrounds included White, African American, Hispanic, Asian, and American Indian or Alaska Native.
Recruitment	Human samples were procured through biobanks and commercial vendors with informed consent and approval under individual Institutional Review Boards. Different Biobank selection was aimed to include samples from diverse sources with blinded information to reduce self-selection bias during the xVFA testing. The established biobanks provided de-identified samples collected under standardized protocols, ensuring consistent quality and ethical compliance.<br><br>Human samples used for training and testing of the xVFA were obtained from the Lyme Disease Biobank. The cohort included 35 two-tier positive Lyme disease samples with signs and symptoms of early Lyme disease. Patient samples in this group were sourced from individuals in Lyme disease-endemic regions, with EM lesions diagnosed by a physician when present, irrespective of size. Participants also included those from endemic areas presenting with symptoms like headache, fatigue, fever, chills, or musculoskeletal pain, or with a history of suspected tick exposure. Additionally, 35 healthy control samples were collected from regions where Lyme disease is endemic, with at least two years of recorded residency in the region. The overarching exclusion criteria for all groups were immunocompromised status, initiation of antibiotics more than 48 hours before sample collection, and age below 10 years. For the LD-positive group, exclusions extended to individuals with reactions to tick bites that did not result in EM or expanding annular lesions and those with a history of chronic fatigue syndrome, rheumatologic diseases, or multiple sclerosis. Further information on Lyme Disease Biobank repository establishment can be found here: PMC7269379.<br><br>The de-identified CDC LD samples in this study included early acute LD-positive samples with localized EM lesions of at least 5 cm. For early disseminated and late-stage LD samples, clinical manifestations were consistent with each condition: varying degrees of heart block for Lyme carditis, specific neurological symptoms for neuroborreliosis, and intermittent or chronic oligoarticular arthritis for late Lyme arthritis. Diagnosis was corroborated wherever possible using culture and/or PCR evidence of B. burgdorferi infection. Patients were excluded if they had significant immunocompromising conditions or if they were unable or unwilling to follow the protocol for sample collection. Additionally, for late Lyme arthritis, patients with a history of certain rheumatological conditions or syphilis were also excluded. All individuals were at least 18 years of age and had appropriate epidemiological risk at the time of collection. Detailed demographic information and sample-specific characterization for these samples are not included in this paper following the CDC's guidance. Further information regarding the CDC's LD samples repository can be found in a previous publication describing the repository development: PMC4187768.
Ethics oversight	De-identified Lyme disease samples were collected from patients with informed consent and the approval of institutional review boards through the Lyme Disease Biobank, under IRB approval from Advarra, protocol Pro00012408. Testing with these de-identified human samples was conducted in accordance with the guidelines of the University of California, Los Angeles's IRB.

Note that full information on the approval of the study protocol must also be provided in the manuscript.

Field-specific reporting

Please select the one below that is the best fit for your research. If you are not sure, read the appropriate sections before making your selection.

☒ Life sciences ☐ Behavioural & social sciences ☐ Ecological, evolutionary & environmental sciences

For a reference copy of the document with all sections, see nature.com/documents/nr-reporting-summary-flat.pdf

Life sciences study design

All studies must disclose on these points even when the disclosure is negative.

Sample size	The samples in this study were acquired based on availability and characterization of the serum using two-tier serology. No sample size calculations were performed.<br><br>The training cohort included 40 of the 70 serum samples procured from Lyme Disease Biobank as described in Sample Cohort 3 of the manuscript. The training subset included 20 early-stage Lyme disease samples and 20 healthy control samples. The validation subset included 30 of the 70 serum samples described in Cohort 3. This included 15 early-stage Lyme disease samples and 15 healthy control samples. All the

samples in Cohort 3 were collected from East Hampton, NY, between 2014 and 2019 (EH), and Marshfield, WI, from 2016 to 2019 (WI)31. Samples were collected through LDB sponsor protocol with Institutional review board (IRB) approval Advarra IRB protocol Pro00012408. Within the LDB sample cohort, there was a nearly even gender distribution, with 48.3% females and 51.3% males. Two-tier positive samples were from patients who either did or did not present erythema migrans (EM) at the time of blood draw. Samples that presented EM lesions were diagnosed by a physician and included in the study irrespective of size. Participants also included those from endemic areas presenting with symptoms like headache, fatigue, fever, chills, or musculoskeletal pain, or with a history of suspected tick exposure. Additionally, the 35 healthy control samples in this cohort were collected from regions where Lyme disease is endemic, with at least two years of recorded residency in the region. The overarching exclusion criteria for all groups were immunocompromise, initiation of antibiotics more than 48 hours before, and age below 10 years. For the LD-positive group, exclusions extended to individuals with reactions to tick bites that did not result in EM or expanding annular lesions and those with a history of chronic fatigue syndrome, rheumatologic diseases, or multiple sclerosis.

The CDC cohort used for assessment of the xVFA included 32 samples representing early and late-stage Lyme disease, potential cross-reactive diseases, and healthy samples from both endemic and non-endemic regions. The CDC LD repository included early acute LD-positive samples with localized EM lesions of at least 5 cm. For early disseminated and late-stage LD samples, clinical manifestations were consistent with each condition: varying degrees of heart block for Lyme carditis, specific neurological symptoms for neuroborreliosis, and intermittent or chronic oligoarticular arthritis for late Lyme arthritis. Diagnosis was corroborated wherever possible using culture and/or PCR evidence of B. burgdorferi infection. Patients were excluded if they had significant immunocompromising conditions or if they were unable or unwilling to follow the protocol for sample collection. Additionally, for late Lyme arthritis, patients with a history of certain rheumatological conditions or syphilis were also excluded. All individuals were at least 18 years of age and had appropriate epidemiological risk at the time of collection. Detailed demographic information and sample specific characterization for these samples are not included in this paper following the CDC's guidance. Further information regarding the CDC's LD samples repository can be found in a previous publication describing the repository's development.

Data exclusions: No data were excluded.

Replication: Each of the samples were tested through three separate xVFA tests.

Randomization: The study includes samples from different biobanks to ensure that the accurate performance of test across different cohort consisting early disease samples and look-alike disease samples previously untested using the diagnostic algorithm.

Blinding: The samples from both Bay Area Lyme Disease Biobank (LDB) and the CDC were received with de-identified and blinded labels. The training and validation cohort samples from LDB were tested using the developed xVFA assay, and subsequently, the results were shared with the Lyme Disease Biobank to reveal the disease classifications. The xVFA predictions for the CDC samples were shared with the CDC after analysis using the trained algorithm to reveal the sample labels.

Reporting for specific materials, systems and methods

We require information from authors about some types of materials, experimental systems and methods used in many studies. Here, indicate whether each material, system or method listed is relevant to your study. If you are not sure if a list item applies to your research, read the appropriate section before selecting a response.

Materials & experimental systems

n/a	Involved in the study	
☐	☒	Antibodies
☒	☐	Eukaryotic cell lines
☒	☐	Palaeontology and archaeology
☒	☐	Animals and other organisms
☒	☐	Clinical data
☒	☐	Dual use research of concern
☒	☐	Plants

Methods

n/a	Involved in the study	
☒	☐	ChIP-seq
☒	☐	Flow cytometry
☒	☐	MRI-based neuroimaging

Antibodies

Antibodies used: Anti-human IgM (#9022-01), IgG (#9040-01) and anti-mouse IgG (#1036-01) were obtained from SourthernBiotech (Birmingham, AL).

Validation: The antibodies were validated for use in our assay during optimization of the assay conditions.
